# Supplementary material for: Diffusion tensor imaging analysis along the perivascular space suggests impaired glymphatic clearance in Lewy body dementia subtypes
Source: Alzheimers Res Ther. 2026 Mar 25;18:101. doi: 10.1186/s13195-026-02021-8 (PMC13137496; doi:10.1186/s13195-026-02021-8)
Supplement: Supplementary file 1 — Supplementary Material 1. [file 13195_2026_2021_MOESM1_ESM.docx]

# Supplementary Materials

## Supplementary Methods

### Approximation of NPI-4

The NPI-4 captures four domains of neuropsychiatric symptoms relevant to LBD. Whilst participants in our study did not complete the NPI-4, they did complete clinical questionnaires relating to each of these domains.

For hallucinations, we used the UMPDHQ, which includes ratings of frequency and severity identical to those used in the NPI-4. For Depression, frequency was approximated using the highest rated item on the PHQ9 or HADS, with the exception of items relating to slowed movements. Depression severity was estimated using the UPDRS item 1.3. Apathy was estimated using the UPDRS item 1.5. As no other measure of apathy was available, the frequency of apathy was assumed to be at least weekly, as this is the time scale asked about during the UPDRS-I questions. Finally, delusions were assumed to be a score of zero for both frequency and severity unless a score of 4 (patient has delusions or paranoia) was given on UPDRS item 1.2. Due to the timescale asked about in UPDRS-I, this was again assumed to occur at least weekly. The scores on each clinical scale and their assumed NPI-4 item scores are detailed below in Supplementary Table 1.

### MRI acquisition

For 8 participants, proton density weighted multiparameter maps were collected using multi-echo fast low angle shot (FLASH) multiparameter mapping scans with proton density weighting were acquired using the following parameters: TR=25ms, eight equidistant echoes with TE1=2.3ms and TE8=18.4ms, flip angle = 6°, bandwidth = 488Hz/pixel, matrix size=224x256x180 with 1mm3 isotropic resolution, acquisition time 9m10s with GRAPPA enabled. Alongside this, an RF sensitivity map was acquired^1^ and an estimate of the B1+ transmit field was calculated at the time of scanning using a 3D Bloch-Siegert based approach ^2^.

These were used to generate synthetic SWI-like images using the CLEAR-SWI pipeline, as described previously ^3^. In brief, muti-echo magnitude and phase data are echo-combined, phase data are unwrapped and background-corrected, magnitude inhomogeneities are removed, and the processed phase is high-pass filtered before combining with magnitude images to produce SWI-like contrast. Filter size was set to [3,3,0].

### Small Vessel Disease Ratings

To quantify each form of cerebral small vessel disease, the T2 and FLAIR scans were rated as follows:

1. White Matter Hyperintensity (WMH).White matter changes were rated on FLAIR images using the modified Fazekas scale^4^. Each longest-diameter white matter change around the lateral ventricles (capping or banding on the periventricular areas) or deep white matter were evaluated separately. For the periventricular WM, a score of 1 represented maximum diameters<5 mm; 2 represented maximum diameters ≥5 mm and <10 mm; and 3 represented maximum diameters≥10 mm. For the Deep WM, a score of 1 represented maximum diameters <10 mm; 2 represented maximum diameters ≥10 mm but <25 mm; and 3 represented maximum diameters ≥25 mm.
2. Cerebral microbleeds (CMBs). MARS visual rating scale was used to count and record CMB locations^5^. CMBs were defined as hypointense areas on SWI, well defined, ovoid or round, and <10mm diameter. The number and location (infratentorial, deep, lobar) of CMBs was recorded.
3. Lacunes of presumed vascular origin. These were visually identified on FLAIR images as hypointense lesions, between 3-15mm in diameter, following STRIVE-2 criteria^6^.
4. Enlarged perivascular spaces (EPVS) were rated in the basal ganglia on T2-weighted MRI following standardised visual rating scale^7^. These were scored as 0 - no EPVS; 1- mild (1-10 EPVS); 2- moderate (11-20 EPVS); 3- frequent (21-40 EPVS); 4- severe (>40EPVS); and midbrain scoring of 0 (No EPVS visible) or 1 (EPVS visible).

Raters had inter-rater agreement ranging from 77 – 97%. For each feature of the small vessel disease total score, the inter-rater agreement was as follows: WMH – 77%, CMB – 87%, lacunes – 94%, EPVS in basal ganglia– 90%, EPVS in midbrain – 97%.

| Supplementary Table 1. Scoring of NPI-4 approximation. | | | |
| --- | --- | --- | --- |
| NPI-4 item | Measure | Item score from alternative measure | Derived NPI-4 item score |
| Hallucinations - Frequency | UMPDHQ - Frequency | 1. *Occasionally* (less than once a week, but continuously) | 1. *Occasionally* – less than once per week |
|  |  | 2. *Often* (about once per week) | 2. *Often* – about once a week |
|  |  | 3. *Frequently* (several times per week but < than once per day) | 3. *Frequently* – several times per week, but less than every day |
|  |  | 4. *Very frequently* (≥once per day) | 4. *Very frequently* – daily or essentially continuously present |
| Hallucinations - Severity | UMPDHQ - Severity | 1. *Mildly* – produce little distress | 1. *Mild* – hallucinations are present but harmless and cause little distress to the patient |
|  |  | 2. *Moderately* – produce distress and are disturbing and disruptive | 2. *Moderate* – hallucinations are distressing and disruptive to the patient |
|  |  | 3. *Severely* – very disturbing (medications may be required) | 3. *Marked* – hallucinations are very disruptive and are a major source of behavioural disturbance. PRN medications may be required to control them. |
| Depression – Frequency | PHQ9 | 1. *Several days* | 2. *Often* – about once a week |
|  |  | 2. *More than half the days* | 3. *Frequently* – several times per week, but less than every day |
|  |  | 3. *Nearly every day* | 4. *Very frequently* – daily or essentially continuously present |
|  | HADS (depression items only) | 1. *Occasionally* (or equivalent) | 1. *Occasionally* – less than once per week |
|  |  | 2. *A lot of the time* (or equivalent) | 2. *Often* – about once a week |
|  |  | 3. *Most of the time* (or equivalent) | 4. *Very frequently* – daily or essentially continuously present |
| Depression - severity | UPDRS item 1.3 | 2. *Mild*: Depressed mood that is sustained over days, but without interference with  normal activities and social interactions. | 1. *Mild* – depression is distressing, but usually responds to redirection or reassurance |
|  |  | 3. *Moderate:* Depressed mood that interferes with, but does not preclude, the patient’s ability to carry out normal activities and social interactions. | 2. *Moderate* – depression is distressing, depressive symptoms are spontaneously voices by the patient and difficult to alleviate. |
|  |  | 4: *Severe:* Depressed mood precludes patients’ ability to carry out normal activities and social interactions | 3. *Marked* – depression is very distressing and a major source of suffering for the patient. |
| Apathy – frequency | UPDRS item 1.4 | 1 - 4 | 2. *Often* – about once a week |
| Apathy – severity | UPDRS item 1.4 | 1. *Slight:* Apathy appreciated by patient and/or caregiver, but no interference with daily activities and social interactions. | 1. *Mild* - apathy is notable but produces little interference with daily routines; only mildly different from patient’s usual behavior; patient responds to suggestions to engage in activities. |
|  |  | 2. *Mild*: Apathy interferes with isolated activities and social interactions.  3. *Moderate*: Apathy interferes with most activities and social interactions. | 2. *Moderate* - apathy is very evident; may be overcome by the caregiver with coaxing and encouragement; responds spontaneously only to powerful events such as visits from close relatives or family members |
|  |  | 4. *Severe*: Passive and withdrawn, complete loss of initiative. | 3. *Marked* - apathy is very evident and usually fails to respond to any encouragement or external events. |
| Delusions – frequency | UPDRS item 1.2 | 4. *Severe:* Patient has delusions or paranoia | 2. *Often* – about once a week |
| Delusions - severity | UPDRS item 1.2 | 4. *Severe:* Patient has delusions or paranoia | 2. *Moderate* - Delusions are distress UMing and disruptive |

GAD7 = Generalised Anxiety Disorder-7, HADS = Hospital Anxiety and Depression Scale, PHQ9 = Patient Health Questionnaire-9, UMPDHQ = University of Miami Parkinson’s Disease Hallucinations Quotient, UPDRS = Unified Parkinson’s Disease Rating Scale

## Supplementary Results

When examining MCI separately to PDD and DLB groups, DTI-ALPS still differed significantly between groups, controlling for age and sex, F(4, 164) = 16.75, p<.0001, as shown in Supplementary Figure 4. DTI-ALPS was significantly reduced for DLB and MCI compared to controls, with a trend towards a reduction in DTI-ALPS for PDD. Relative to PD-NC, DTI-ALPs was also reduced in all cognitively impaired groups (MCI, PDD and DLB). DTI-ALPS was also further reduced in DLB compared to PDD and MCI.

When further separating MCI into PD-MCI and MCI-LB, controlling for age and sex, the main pattern of differences remained, F(4, 138) = 16.06, p<.0001. Importantly, there was no difference in DTI-ALPS between PD-MCI and PDD groups, nor between MCI-LB and DLB groups, supporting the grouping of these for the main analyses (Supplementary Figure 5). Additionally, there were no significant differences in DTI-ALPS between PD-MCI and MCI-LB.

Average complexity also differed significantly between groups, F(4, 161) = 9.75, p<.0001, as shown in Supplementary Figure 6. Average complexity was significantly reduced for DLB and MCI compared to controls, with a trend towards reduced complexity in PDD relative to controls. Relative to PD-NC, average complexity was reduced in all cognitively impaired groups (MCI, PDD, DLB). There were no differences in average complexity between MCI, PDD and DLB, nor between PD-NC and controls. When further separating MCI into PD-MCI and MCI-LB, the pattern of results remained the same, F(4, 135) = 7.87, p<.0001, Supplementary Figure 7

Examining MCI separately to PDD and DLB groups, controlling for average complexity, age and sex, DTI-ALPS still differed between groups F(4, 160) = 20.49, p<.0001. There was no change to the pattern of results, a reduction in DTI-ALPS for DLB and MCI compared to control and a trend towards reduced DTI-ALPS in PDD relative to controls. Relative to PD-NC, DTI-ALPs was reduced in all groups with cognitive involvement (MCI, PDD, DLB). DTI-ALPS was also reduced in DLB compared to PDD and MCI (Supplementary Figure 4). When further separating MCI into PD-MCI and MCI-LB, and controlling for age, sex and complexity, there was also no change to the pattern of results, F(4, 134) = 20.31, p<.0001, Supplementary Figure 5.

**Supplementary Table 2. Demographic and clinical information, when separating patients with MCI**

|  |  | | Controls  (n = 26) | PD  (n = 60) | MCI  (n = 21) | PDD  (n = 37) | DLB  (n = 50) | Test statistic | p-value |
| --- | --- | --- | --- | --- | --- | --- | --- | --- | --- |
|  | Age | | 66.7 (9.28) | 63.1 (7.33) ^a^ | 67.9 (7.81) ^b^ | 71.9 (7.00) ^b^ | 72.5 (5.75) ^a,b,c^ | χ² = 43.8 | **<.0001** |
|  | Gender | | 13 M / 13 F | 27 M / 33 F | 13 M / 7 F | 13 M / 3 F ^b^ | 42 M / 8 F ^a,b^ | χ² = 21.9 | **<.0001** |
|  | Years Education | | 17.4 (2.9) | 16.3 (2.6) | 15.6 (2.9) | 16.0 (3.6) | 15.0 (3.2) | χ² =8.1 | .09 |
|  | |  | | | | | | | |
|  | UPDRS-III | | 5.65 (4.8) | 19.9 (10.1) ^a^ | 21.9 (9.0) ^a^ | 39.1 (12.9) ^a,b,c^ | 37.1 (17.5) ^a,b,c^ | χ² = 81.9 | **<.0001** |
|  | UPDRS-total | | 9.3 (5.9) | 40.3 (18.7) ^a^ | 38.3 (16.0) ^a^ | 79.4 (21.9) ^a,b,c^ | 72.8 (28.7) ^a,b,c^ | χ² = 105.2 | **<.0001** |
|  | LEDD | | - | 473 (282) | 604 (256) | 669 (398) ^b^ | 239 (250) ^b,c,d^ | χ² = 39.0 | **<.0001** |
|  | Disease Duration Parkinsonism | | - | 5.9 (14.2) | 6.4 (4.7) | 7.2 (3.9) ^b^ | 2.1 (2.2) ^b,c,d^ | χ² = 51.4 | **<.0001** |
|  | Disease Duration Cognition | | - | - | 0.5 (1.6) | 2.0 (2.1) ^c^ | 2.1 (2.2) ^c^ | χ² = 21.0 | **<.0001** |
|  | |  | | | | | | | |
|  | MMSE | | 29.1 (1.0) | 29.3 (0.8) | 28.3 (1.3) ^a,b^ | 26.9 (3.2) ^a,b^ | 24.4 (4.0) ^a,b,c,d^ | χ² = 81.1 | **<.0001** |
|  | MOCA | | 28.8 (1.3) | 28.7 (1.2) | 27.0 (2.1) ^a,b^ | 21.6 (4.1) ^a,b,c^ | 20.5 (5.2) ^a,b,c^ | χ² = 113.2 | **<.0001** |
|  | Composite Cognitive Score | | 0.02 (0.6) | -0.01 (0.6) | -0.80 (0.8) ^a,b^ | -2.09 (1.5) ^a,b,c^ | -2.90 (1.7) ^a,b,c^ | χ² = 90.5 | **<.0001** |

All values are mean(standard deviation), apart from gender which is shown as a proportion; DLB= Dementia with Lewy bodies; MMSE = Mini Mental State Examination, MoCA = Montreal Cognitive Assessment, LEDD = Levodopa equivalent daily dose, PD = Parkinson’s disease; PDD = Parkinson’s Dementia; UPDRS-III = Unified Parkinson’s Disease Rating Scale part 3 (motor assessment). For people with PDD, disease duration is specified separately for onset of motor Parkinsonism and onset of dementia. Significant differences between groups are ^a^ Significantly different from controls (for all other groups). ^b^ significantly different from PD (PDD and DLB). ^c^ significantly different from MCI (for PDD and DLB). ^d^ significantly different from PDD (for DLB only)

**Supplementary table 3. Cognitive and questionnaire scores when separating patients with MCI**

|  |  | Controls  (n = 26) | PD  (n = 60) | MCI  (n = 21) | PDD  (n = 16) | DLB  (n = 52) | Test statistic | FDR corrected p-value |
| --- | --- | --- | --- | --- | --- | --- | --- | --- |
| Cognitive measures | |  |  |  |  |  |  |  |
|  | Digit span backwards | 7.4 (2.2) | 7.5 (2.2) | 8.1 (2.3) | 5.3 (1.7) ^a,b,c^ | 5.4 (1.7) ^a,b,c^ | χ² = 21.7 | **.0002** |
|  | Stroop colour naming (s) | 32.1 (6.3) | 32.0 (6.6) | 35.6 (10.7) | 52.3 (14.9) ^a,b,c^ | 52.4 (17.0) ^a,b,c^ | χ² = 71.4 | **<.0001** |
|  | Stroop interference (s) | 54.7 (9.9) | 58.4 (13.1) | 64.1 (18.8) | 127.2 (67.7) ^a,b,c^ | 124.9 (76.8) ^a,b,c^ | χ² = 82.9 | **<.0001** |
|  | Verbal fluency category | 21.6 (7.8) | 21.6 (6.5) | 19.3 (5.9) | 10.8 (4.9) ^a,b,c^ | 10.4 (4.8) ^a,b,c^ | χ² = 82.4 | **<.0001** |
|  | Graded naming task | 23.9 (5.0) | 23.9 (3.3) | 23.2 (5.1) | 19.0 (4.8) ^a,b,c^ | 20.2 (5.6) ^a,b,c^ | χ² = 25.9 | **<.0001** |
|  | Verbal fluency letter | 17.7 (6.2) | 17.6 (5.2) | 16.4 (6.1) | 13.1 (4.9) ^b^ | 10.5 (5.0) ^a,b,c^ | F = 14.6 | **<.0001** |
|  | Word recognition task | 24.5 (1.1) | 24.4 (0.9) | 23.3 (1.6) ^a,b^ | 21.3 (6.7) ^a^ | 21.3 (3.3) ^a,b^ | χ² = 40.3 | **<.0001** |
|  | Logical memory (delayed) | 14.6 (4.4) | 14.3 (3.8) | 11.0 (3.6) ^b^ | 8.0 (6.1) ^a,b^ | 6.6 (4.4) ^a,b,c^ | χ² = 49.7 | **<.0001** |
|  | Hooper Visual Organisation | 25.7 (2.1) | 25.0 (3.1) | 22.4 (3.2) ^a,b^ | 12.5 (6.2) ^a,b,c^ | 16.7 (6.0) ^a,b,c^ | χ² = 86.7 | **<.0001** |
| Clinical Questionnaires | |  |  |  |  |  |  |  |
|  | HADS Depression Score | 1.3 (1.5) | 3.5 (2.7) ^a^ | 4.5 (2.9) ^a^ | 8.1 (4.8) ^a,b,c^ | 6.0 (3.1) ^a,b^ | χ² = 51.7 | **<.0001** |
|  | HADS Anxiety Score | 3.7 (3.6) | 5.6 (2.6) ^a^ | 5.1 (3.8) | 9.2 (6.3) ^a^ | 5.6 (3.6) ^a^ | χ² = 11.3 | .069 |
|  | Hallucinations (UMPDHQ) | 0 (0) | 0.7 (1.9) | 1.2 (2.2) | 2.1 (3.7) ^a^ | 4.1 (3.3) ^a,b,c,d^ | χ² = 61.1 | **<.0001** |
|  | NPI-4 | - | 1.4 (2.3) | 1.9 (2.5) | 5.4 (5.2) ^b,c^ | 5.6 (4.4) ^b,c^ | χ² = 28.9 | **<.0001** |
|  | Sleep (RBDSQ) | 2.2 (1.4) | 4.3 (2.4) ^a^ | 5.8 (4.0) ^a^ | 6.3 (3.1) ^a^ | 7.9 (3.8) ^a,b,c^ | χ² = 46.6 | **<.0001** |
|  | Fluctuations (CAF) |  | - | - | 4.8 (3.5) | 6.7 (4.1) ^d^ | W = 137.5 | .45 |
|  | Fluctuations (One-Day) |  | - | - | 8.0 (6.3) | 7.6 (5.6) | W = 115 | .96 |
|  | Fluctuations (DCFS) |  | - | - | 9.2 (2.5) | 9.4 (4.4) | W = 125.5 | .96 |
|  | Functional activities |  | - | 2.8 (3.2) | 12.9 (6.9) ^c^ | 11.6 (6.9) ^c^ | χ² = 22.7 | **.0002** |
|  | LBD symptom composite |  | - | - | 31.8 (4.5) | 25.4 (7.6) ^d^ | W = 49.5 | .068 |

All values are mean(standard deviation). CAF=Clinician Assessment of Fluctuations; DLB= Dementia with Lewy bodies; HADS=Hospital Anxiety and Depression Scale; MCI = Mild cognitive impairment (either PD-MCI or MCI-LB); PD= Parkinson’s disease; PDD = Parkinson’s Dementia; RBDSQ=REM Sleep Behaviour Disorder Questionnaire. Significant differences between groups are ^a^ Significantly different from controls (for all other groups). ^b^ significantly different from PD (for MCI, PDD and DLB). ^c^ significantly different from MCI (for PDD and DLB). ^d^ significantly different from PDD (for DLB group only)

**Supplementary Figure 1**. **Association between DTI-ALPS and average complexity.** DTI-ALPS = diffusion tensor imaging along the perivascular space. Both DTI-ALPS and complexity metric use arbitrary units.
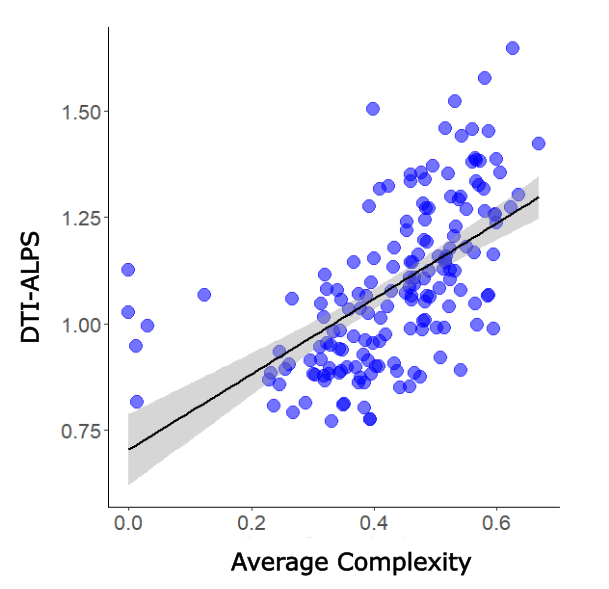


**Supplementary Figure 2. Differences in average white matter complexity between patients with Parkinson’s disease without cognitive involvement, Parkinson’s disease dementia and dementia with Lewy bodies.** PD=Parkinson’s Disease, DLB= dementia with Lewy bodies, MCI = mild cognitive impairment, PDD=Parkinson’s Disease Dementia


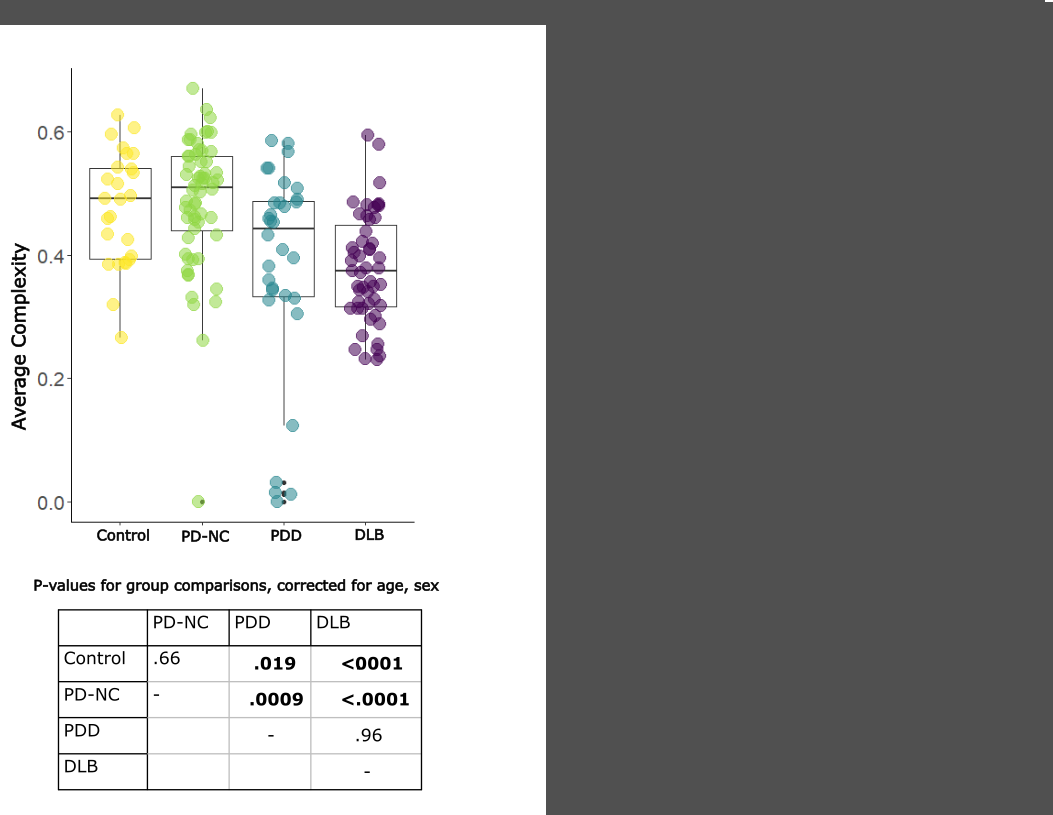


**Supplementary Figure 3. Associations between complexity of the Superior Longitudinal Fasciculus and complexity of the Superior Corona Radiata in each hemisphere**. SLF = superior longitudinal fasiculus, SCR = superior corona radiata.
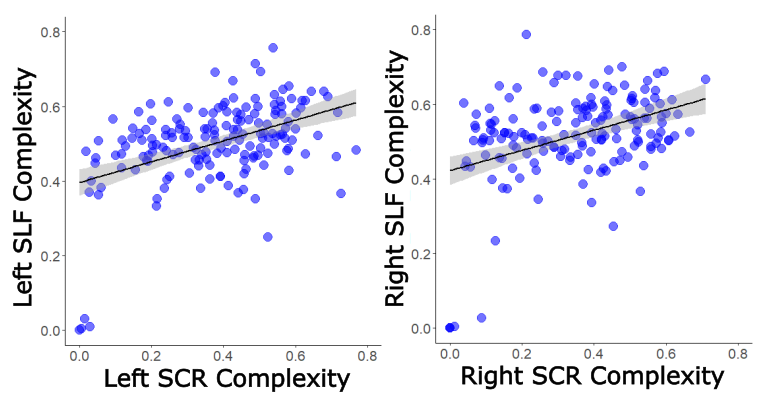


**Supplementary Figure 4. Differences in DTI-ALPS between controls, patients with Parkinson’s disease without cognitive involvement, Mild Cognitive Impairment, Parkinson’s disease dementia and dementia with Lewy bodies.** PD=Parkinson’s Disease, DLB= dementia with Lewy bodies, MCI = mild cognitive impairment, PDD=Parkinson’s Disease Dementia

**
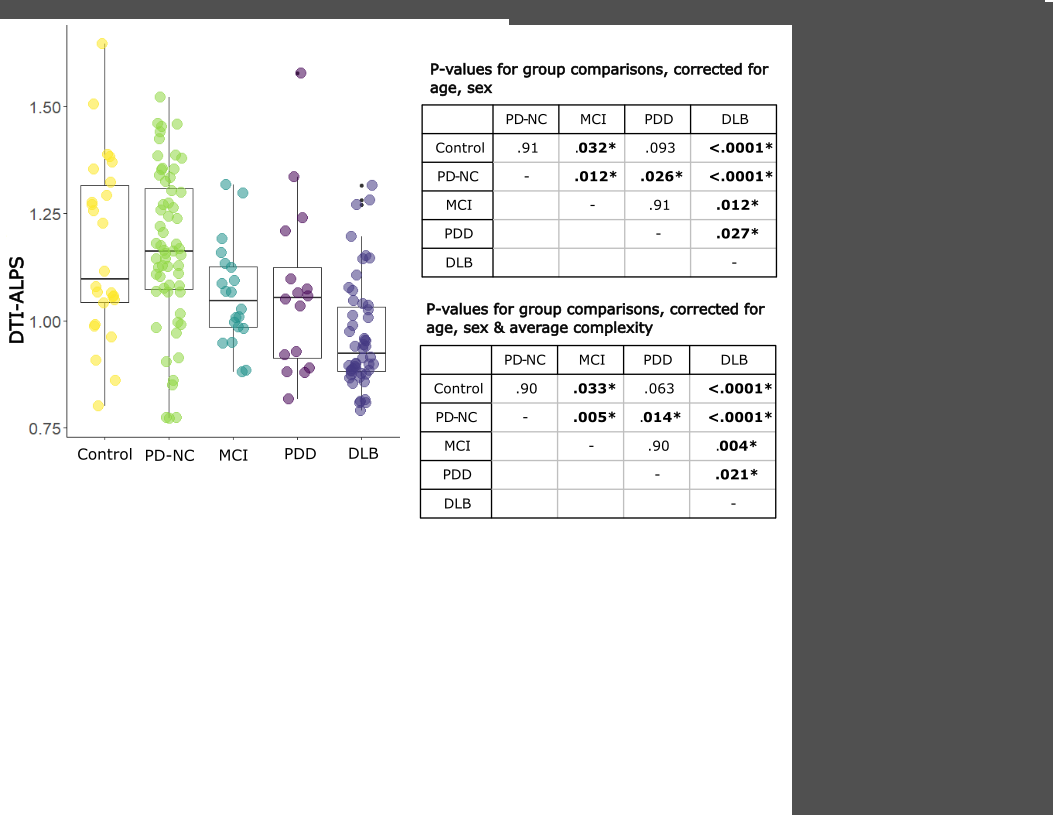
**

**Supplementary Figure 5. Differences in DTI-ALPS between controls, PD-NC, PD-MCI, MCI-LB, PDD and DLB.** PD=Parkinson’s Disease, DLB= dementia with Lewy bodies, MCI-LB = mild cognitive impairment with Lewy bodies, PD-MCI = Parkinson’s disease with mild cognitive impairment, PDD=Parkinson’s Disease Dementia

**
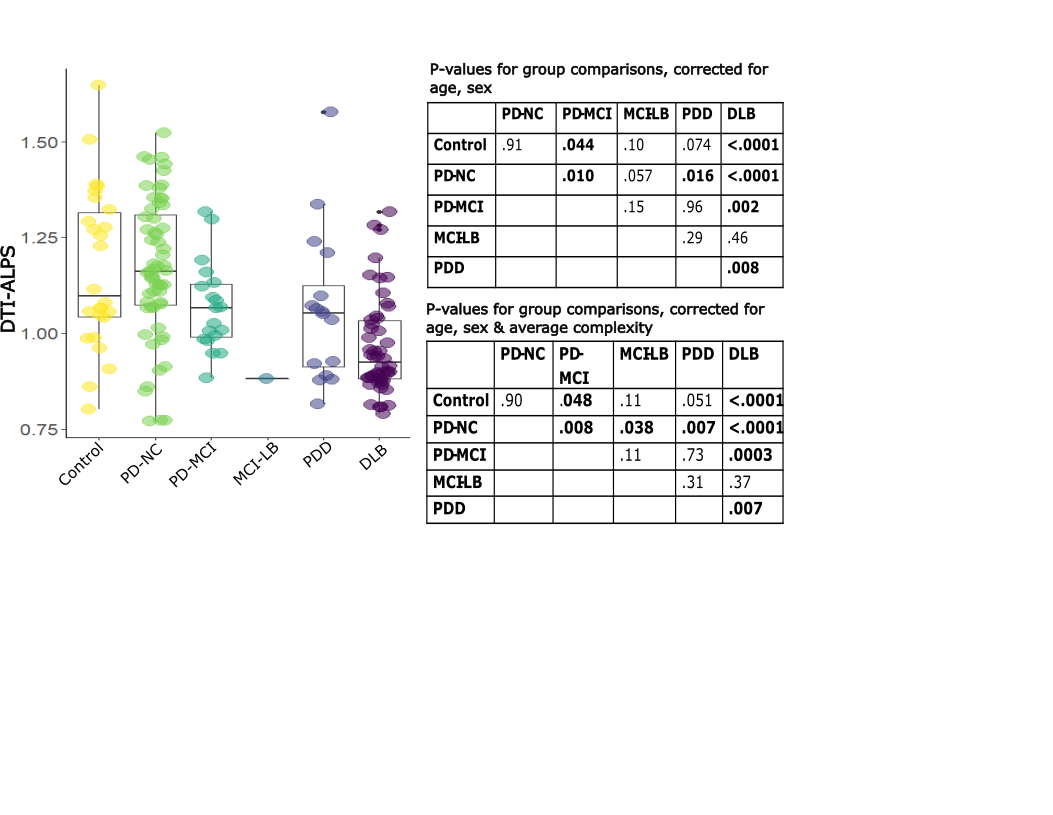

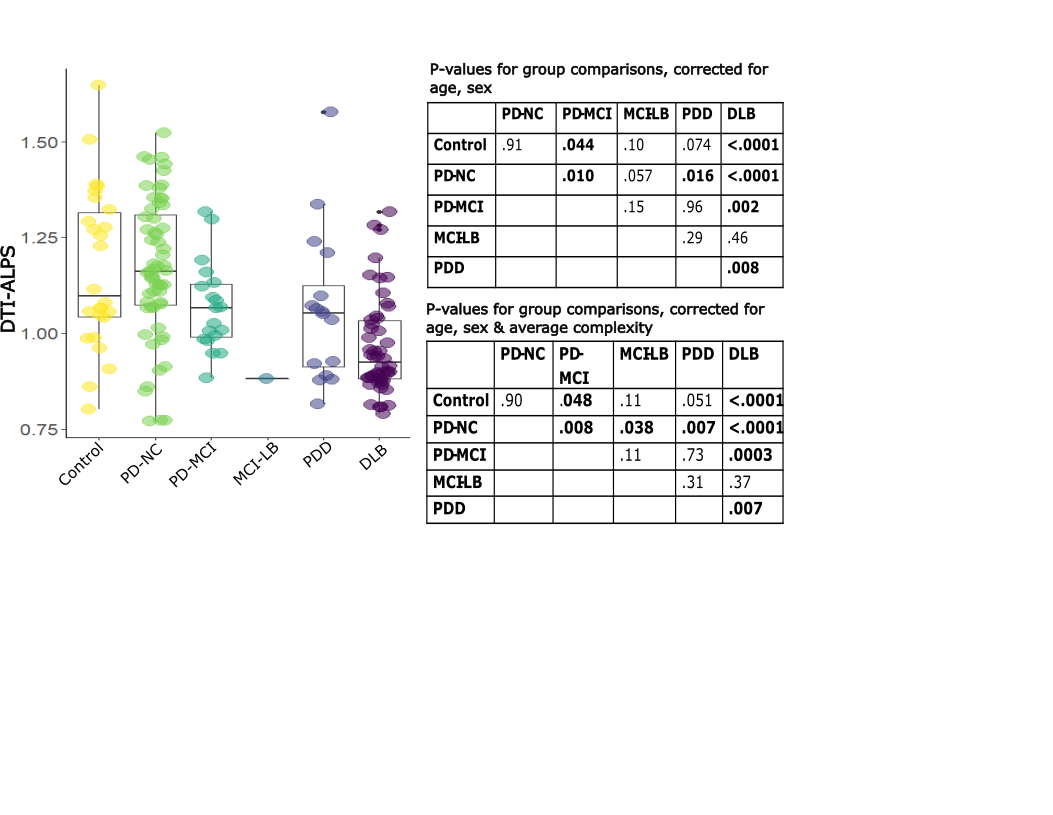
**

**Supplementary Figure 6. Differences in average complexity between controls, patients with Parkinson’s disease without cognitive involvement, Mild Cognitive Impairment, Parkinson’s disease dementia and dementia with Lewy bodies.** PD=Parkinson’s Disease, DLB= dementia with Lewy bodies, MCI = mild cognitive impairment, PDD=Parkinson’s Disease Dementia


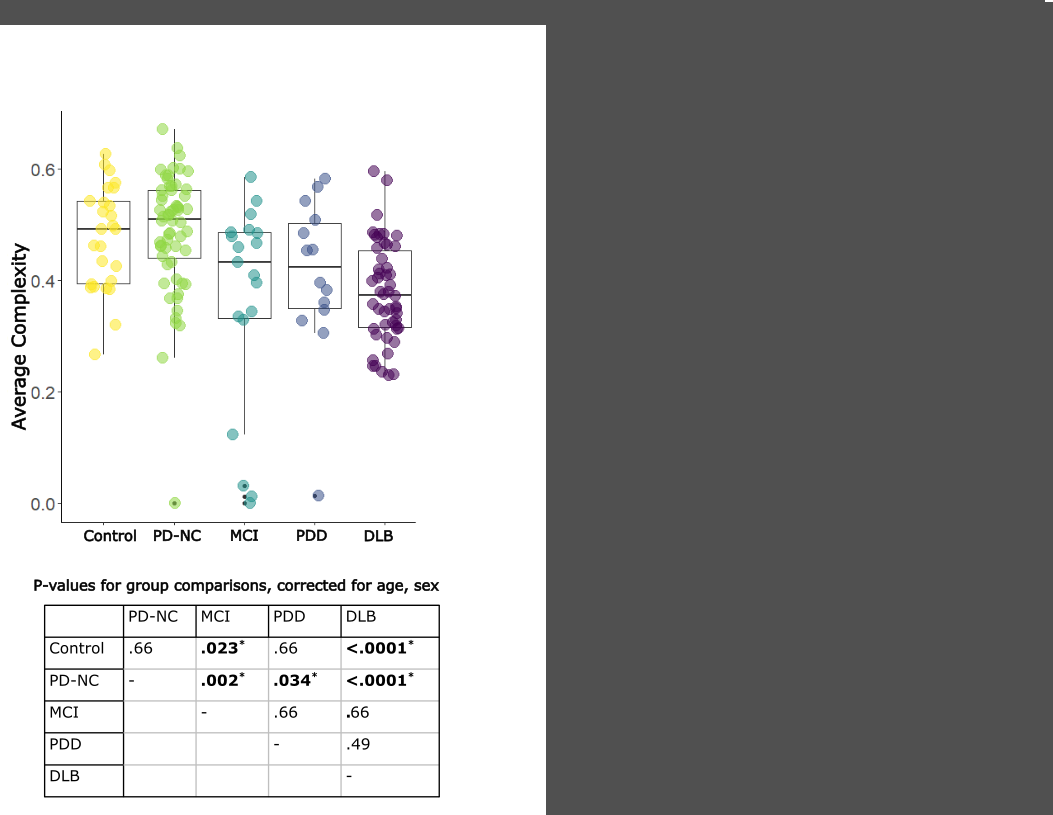


**Supplementary Figure 7. Differences in average complexity controls, PD-NC, PD-MCI, MCI-LB, PDD and DLB.** PD=Parkinson’s Disease, DLB= dementia with Lewy bodies, MCI-LB = mild cognitive impairment with Lewy bodies, PD-MCI = Parkinson’s disease with mild cognitive impairment, PDD=Parkinson’s Disease Dementia

**
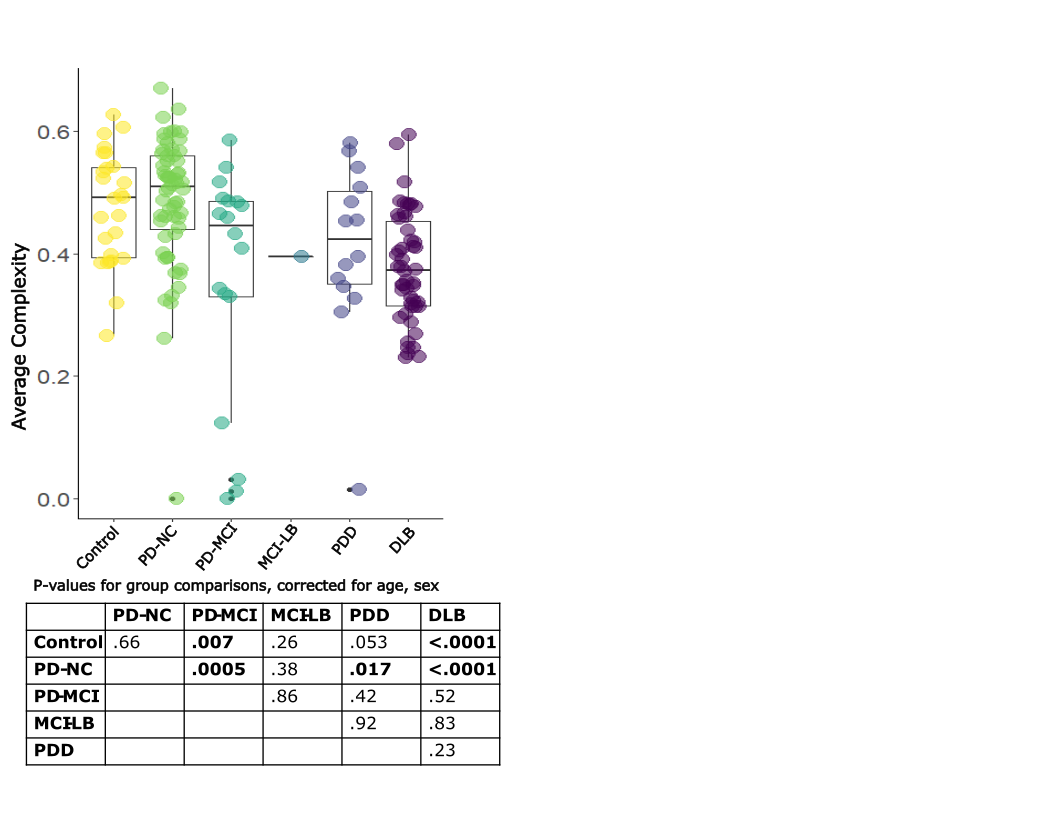
**

**Supplementary Figure 8. Association between DTI-ALPS and disease duration.** Relationship between DTI-ALPS, an indirect measure of glymphatic clearance and disease duration (from onset of first symptom)

Color of dot indicates clinical group: green = PD-NC, blue = PDD, purple = DLB. DLB = Dementia with Lewy Bodies, PD-NC = Parkinson’s disease with normal cognition, PDD = Parkinson’s disease Dementia. DTI-ALPS uses arbitrary units. The raw data for DTI-ALPS and cognitive scores are plotted in the figure; statistical analyses are reported as simple linear regressions and were repeated including age as a covariate and including age, sex and average complexity as covariates. Grey shaded areas represented 95% confidence intervals

**
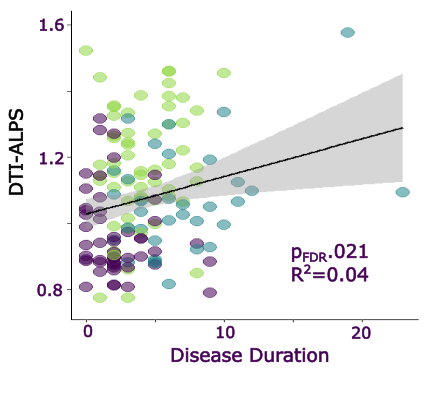
**

Supplementary table 4. **Associations of DTI-ALPS with clinical and cognitive measures, controlling for age, sex and average complexity.**

|  |  | x ~ DTI-ALPS + Age + Sex + Average Complexity | | | |  |
| --- | --- | --- | --- | --- | --- | --- |
|  |  | β DTI-ALPS | FDR corrected p-value DTI-ALPS | VIF DTI-ALPS | Model R² |  |
| Cognitive measures | |  |  |  |  |  |
|  | MMSE | -1.24 | .78 | 2.31 | 0.20 |  |
|  | MOCA | -0.14 | .96 | 2.32 | 0.21 |  |
|  | Composite cognitive score | 0.54 | .78 | 2.44 | 0.19 |  |
| Other clinical measures | |  |  |  |  |  |
|  | UPDRS-III | -6.40 | .78 | 2.34 | 0.09 |  |
|  | UPDRS-total | -11.0 | .78 | 2.35 | 0.07 |  |
|  | Disease Duration | 4.32 | .30 | 2.30 | 0.06 |  |
|  | Hallucinations | -2.36 | .67 | 2.34 | 0.05 |  |
|  | Sleep (RBDSQ) | -2.51 | .67 | 2.33 | 0.12 |  |
|  | CAF (fluctuations)+ | 8.59 | .65 | 2.54 | 0.00 |  |
|  | One-Day Fluctuations+ | 17.25 | .30 | 2.64 | 0.06 |  |
|  | DCFS (fluctuations)+ | 12.6 | .30 | 2.64 | 0.02 |  |
|  | Compass-21 (autonomic) | 2.56 | .90 | 1.86 | 0.00 |  |
|  | NPI-4 | 1.36 | .78 | 2.36 | 0.05 |  |
|  | LBD symptom score+ | 18.5 | .65 | 2.79 | 0.00 |  |
| Plasma measures | |  |  |  |  |  |
|  | p-tau217 | 0.08 | .79 | 2.48 | 0.11 |  |
|  | NfL | -3.39 | .79 | 2.18 | 0.23 |  |
|  | GFAP | -77.3 | .67 | 2.27 | 0.23 |  |
| Vascular measures | |  |  |  |  |  |
|  | History of vascular risk factors | -0.45 | .78 | 2.32 | 0.03 |  |
|  | Total SVD score+ | -1.13 | .83 | 2.54 | 0.00 |  |

+ only available in a subset of LBD patients. CAF=Clinician Assessment of Fluctuations; DCFS = Dementia Cognitive Fluctuations Scale; GFAP = Glial fibrillary acidic protein; HADS=Hospital Anxiety and Depression Scale; LBD = Lewy Body Dementia; MMSE = Mini Mental State Examination; MoCA = Montreal Cognitive Assessment; NfL = neurofilament light chain; NPI-4 = Neuropsychiatric Inventory; RBDSQ=REM Sleep Behaviour Disorder Questionnaire; SVD = small vessel disease; UMPDHQ = University of Miami Parkinson’s Disease Hallucinations Quotient; UPDRS-III = Unified Parkinson’s Disease Rating Scale part 3 (motor assessment); UPDRS-total = Unified Parkinson’s Disease Rating Scale total symptom score.

References

1. Balbastre Y, Aghaeifar A, Corbin N, Brudfors M, Ashburner J, Callaghan MF. Correcting inter‐scan motion artifacts in quantitative R1 mapping at 7T. *Magnetic Resonance in Medicine.* 2022;88(1):280-291.

2. Corbin N, Acosta‐Cabronero J, Malik SJ, Callaghan MF. Robust 3D Bloch‐Siegert based mapping using multi‐echo general linear modeling. *Magnetic resonance in medicine.* 2019;82(6):2003-2015.

3. Eckstein K, Bachrata B, Hangel G, et al. Improved susceptibility weighted imaging at ultra-high field using bipolar multi-echo acquisition and optimized image processing: CLEAR-SWI. *Neuroimage.* 2021;237:118175.

4. Rieu Z, Kim RE, Lee M, et al. A fully automated visual grading system for white matter hyperintensities of T2-fluid attenuated inversion recovery magnetic resonance imaging. *Journal of Integrative Neuroscience.* 2023;22(3):57.

5. Gregoire S, Chaudhary U, Brown M, et al. The microbleed anatomical rating scale (MARS) reliability of a tool to map brain microbleeds. *Neurology.* 2009;73(21):1759-1766.

6. Duering M, Biessels GJ, Brodtmann A, et al. Neuroimaging standards for research into small vessel disease—advances since 2013. *The Lancet Neurology.* 2023;22(7):602-618.

7. Potter G, Morris Z, Wardlaw J. Enlarged perivascular spaces (EPVS): a visual rating scale and user guide. *Guide prepared by Gillian Potter, Zoe Morris and Prof Joanna Wardlaw (University of Edinburgh).* 2015.
